# Influence of Lipidation Pattern of the KR12 Fragment of Peptide LL-37 on Its Antibacterial and Hemolytic Activities

**DOI:** 10.3390/ijms24065505

**Published:** 2023-03-13

**Authors:** Elżbieta Kamysz, Emilia Sikorska, Marta Bauer, Karol Sikora, Damian Neubauer

**Affiliations:** 1 Laboratory of Chemistry of Biological Macromolecules, Department of Molecular Biotechnology, Faculty of Chemistry, University of Gdańsk, 80-308 Gdańsk, Poland; 2 Laboratory of Structural Studies of Biopolymers, Department of Organic Chemistry, Faculty of Chemistry, University of Gdańsk, 80-308 Gdańsk, Poland; 3 Department of Inorganic Chemistry, Faculty of Pharmacy, Medical University of Gdańsk, 80-210 Gdańsk, Poland

**Keywords:** ESKAPE pathogens, *Staphylococcus aureus*, KR12, LL37, lipopeptide, CD spectroscopy, dynamic light scattering

## Abstract

Contemporary medicine has been confronted by multidrug resistance. Therefore, new antibiotics are sought to alleviate the problem. In this study, we estimated the effect of the positioning and extent of lipidation (mainly octanoic acid residue) in the KR12-NH_2_ molecule on antibacterial and hemolytic activities. The effect of the conjugation of benzoic acid derivatives (C_6_H_5_-X-COOH, where X: CH_2_, CH_2_-CH_2_, CH=CH, C≡C, and CH_2_-CH_2_-CH_2_) with the *N*-terminal part of KR12-NH_2_ on biological activity was also studied. All analogs were tested against planktonic cells of ESKAPE bacteria and reference strains of *Staphylococcus aureus*. The effect of lipidation site on the helicity of the KR12-NH_2_ analogs was studied using CD spectroscopy. The ability of the selected peptides to induce the aggregation of POPG liposomes was evaluated with DLS measurements. We demonstrated that both the site and extent of peptide lipidation play an essential role in the bacterial specificity of the lipopeptides. Most of the C_8_^α^-KR12-NH_2_ (**II**) analogs that were more hydrophobic than the parent compound were also more hemolytic. A similar relationship was also found between the α-helical structure content in POPC and hemolytic activity. It is worth emphasizing that in our study, the highest selectivity against *S. aureus* strains with an SI value of at least 21.11 exhibited peptide **XII** obtained by the conjugation of the octanoic acid with the *N*-terminus of retro-KR12-NH_2_. All lipidated analogs with the highest net charge (+5) were the most selective toward pathogens. Therefore, the overall charge of KR12-NH_2_ analogs plays pivotal role in their biological activity.

## 1. Introduction 

One of the most relevant human threats is multidrug resistance and the concomitant high morbidity and mortality rate. In 2019, the most prominent drug-resistant pathogens (*Escherichia coli*, *Staphylococcus aureus*, *Klebsiella pneumoniae*, *Streptococcus pneumoniae*, *Acinetobacter baumannii*, and *Pseudomonas aeruginosa*) caused approximately 3,570,000 infections, including 929,000 fatalities [1]. Therefore, it is crucial to develop new effective antimicrobial agents. Indeed, the WHO emphasizes that R&D should focus on the search and modification of new antimicrobials, especially those against *A. baumannii*, *P. aeruginosa*, *K. pneumoniae*, *Enterobacter* spp., *Enterococcus faecium*, and *methicillin-resistant S. aureus* [2].

The class of compounds that has attracted increasing attention in recent decades as antimicrobial drug candidates or templates for their design is antimicrobial peptides (AMPs), important effectors of innate immunity. Many of them do not target any specific receptor but cause nonmediated membrane destruction which leads to cell lysis and, ultimately, death [3]. This mechanism of action of AMPs is their undisputed advantage because it means that bacteria need several hundred generations at a low concentration of antimicrobial peptides to achieve resistance [4]. There is also evidence that AMPs can enter a bacterial cell to interact with intracellular targets by binding to DNA or RNA or promoting the production of reactive oxygen species to cause further damage. However, the therapeutic efficacy of many AMPs is limited due to a number of factors, including susceptibility to protease degradation, systemic toxicity, and low bioavailability.

One of the valuable methods to increase the antibacterial activity of AMPs is lipidation [3,4,5,6,7,8,9]. The lipophilic entity is usually attached to the *N*-terminus of the AMP, leading to an amide-bond-linked lipidated AMP. The presence of a lipid group in peptides can modulate their secondary structure and increase their affinity to the bilayer. Additionally, AMP lipidation improves their plasma stability either through albumin binding or the formation of supramolecular structures [10]. However, the attachment of fatty acid chains to peptides can also have a negative influence on their membrane-binding properties due to enhanced aggregation propensity, which is highly dependent on the acyl chain length and the reduced effective monomer concentration [11]. The specificity of the lipoAMPs toward different cell types depends on both the size of the aliphatic chain and the length and composition of the peptide sequence [11]. A serious disadvantage of AMP lipidation is that increased length in the acyl chain lowers their solubility in water and selectivity for bacterial and eukaryotic cell membranes. Overcoming this obstacle requires finding an optimal length of fatty acid chains with increased antimicrobial activity but leaving the toxicity towards mammalian cells unaffected.

In our previous research, we showed that the modification of the LL-37-derived peptide fragment KR12-NH_2_ with a lipophilic entity (variable-length fatty acids or aromatic acids) in the *N*-terminal part of the molecule was an effective way to increase its antibacterial activity [12]. The KR12-NH_2_ analog with octanoic acid (**II**) exhibited the highest potency against all tested microorganisms. In this article, we studied the effect of the degree and positioning of the lipophilic entity (mainly the octanoic acid residue) in KR12-NH_2_ (*N*- and the *C*-terminal part of the molecule, the *N*- and *C*-terminal part of the retro-molecule, the side chain amino group of Lys in the middle of KR12-NH_2_, and the side chain amino group of the additional Lys residue coupled with the *N*- and *C*-terminal part of the molecule) on the antibacterial and hemolytic activity. As reported by Albada et al., the switching of lipidation from the *N*-terminal lysine side chain of peptide (RW)_3_ to its *C*-terminal lysine side chain resulted in a reduction in the hemolysis of human red blood cells (hRBCs) from 50 to 10–20% [13]. In this study, we also synthesized conjugates of KR12-NH_2_ with benzoic acids and its longer aromatic derivatives (C_6_H_5_-X-COOH, where X: CH_2_, CH_2_-CH_2_, CH=CH, C≡C, and CH_2_-CH_2_-CH_2_) covalently attached to the *N*-terminal part of the molecule to study the impact of the distance between the phenyl ring and the carboxylic group, as well as carbon–carbon bond multiplicity, on their properties. Modifications with aromatic acids were also dealt with because our previous experiments showed that conjugation with benzoic acid or trans-cinnamic acid of the KR12-NH_2_ peptide led to compounds with promising antibacterial activity and low toxicity [12]. In addition, the KR12-NH_2_ analog modified with 4-phenylbenzoic acid was included to find out whether increasing the number of phenyl rings in the lipophilic entity would improve the usefulness of the peptides as antimicrobials. Studies on short cationic lipopeptides indicate that compounds with branched aliphatic fatty acids have elevated selectivity to pathogens over human cells [14,15] as compared to that of the straight chain ones with similar hydrophobicity. Hence, 2-ethylhexanoic and 2-butyloctanoic acids were used. The effect of inserted modifications on the helicity of the KR-12-NH_2_ peptide was studied in anionic and zwitterionic membranes with CD spectroscopy. The ability of the selected peptides to neutralize and induce the aggregation of POPG liposomes was evaluated using zeta potential and dynamic light scattering (DLS) measurements.

## 2. Results and Discussion

### 2.1. Peptide Synthesis, Purification, and Hydrophobicity

The identity of the purified peptides was confirmed using electrospray ionization mass spectrometry (ESI-MS) in positive ion mode and the results are shown in Table S1 (Supplementary Materials). The sequences, the net charge, the number of carbon atoms in the lipophilic entity, as well as the adjusted retention time of the peptides are displayed in Table 1.

**Figure 1:**
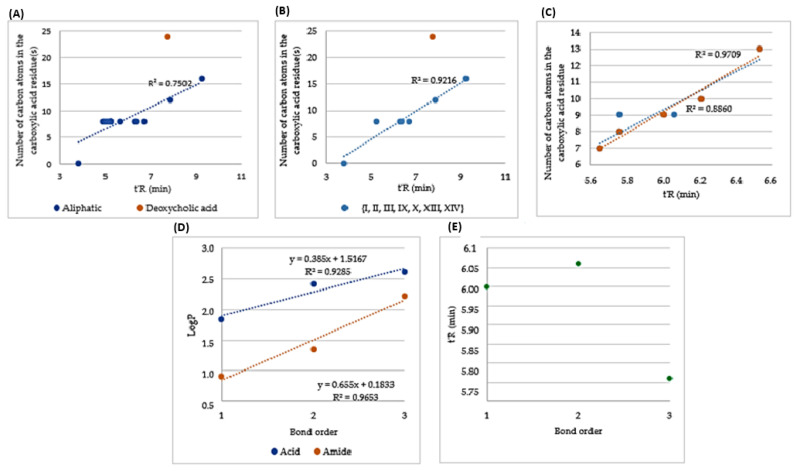
(**A**–**C**) Adjusted retention time (t’_R_) of KR12-NH_2_ analogs with aliphatic carboxylic acid residues ((**A**) **I**–**XIV**; (**B**)—The analogs (**I**, **II**, **III**, **IX**, **X**, **XIII**, and **XIV**); (**C**) The analogs **XV**–**XXI**) vs. the number of carbon atoms in carboxylic acid residue. (**D**) Bond order of aromatic carboxylic acids and amides vs. LogP; (**E**) bond order in aromatic carboxylic acid residues in KR12-NH_2_ analogs vs. t’_R_.

The conjugation of KR12-NH_2_ with aliphatic and aromatic acids affords more hydrophobic compounds than the parent molecule, but it depends on the modified amino group involved. The conjugation of C_8_ to *N*^α^ amino group of the *N*-terminal amino acid gives a more distinct increase in hydrophobicity than that of C_8_ in the sidechain of lysine residues (i.e., **II** vs. **III**; **IV** vs. **V**). The basicity of both lysine amino groups is comparable (pKa *N^α^* = 8.95, pKa *N^ε^* = 10.53). Moreover, in the acidic conditions of the mobile phase (0.1% TFA; pH 2,) both amino groups are protonated. Hypothetically, the acylation of the *N^α^*-amino group disrupts the helix macrodipole and eliminates excessive positive charge [16]. The dipole moment and positive charge can obviously contribute to peptide hydrophilicity. This phenomenon applies exclusively to the *N^α^*-amino group, but not so much to *N^ε^*. It seems that the position of the lysine residue with the modified *N*^ε^_-_amino group is not essential for the observed retention behavior (**III** vs. **VIII**). Similarly, the additional Lys^ε^(C_8_) residue, whether at the *N*- or *C*-terminal position, result in analogs of comparative retention times (**V** vs. **VI**). Two of the analogs have fatty acids attached to both *N^α^*- and *N^ε^*-amino groups (**IX** and **X**). The hydrophobicity of the analog with two butyric acid residues (**X**) is lower than that of analog **II** (C_8_ at *N^α^*), but still higher than that of analog **III** (C_8_ at *N^ε^*). These results, once again, suggest that the coupling of fatty acid with the *N^α^* amino group gives a more distinct change in the retention behavior than that of the acylation of *N^ε^*. Previous studies revealed that a change in the hydrophobicity of the retro-analogs can affect the antimicrobial activity. It was hypothesized that *N*- and *C*-terminal amino acid residues had an essential contribution to the difference between the hydrophobicity of the peptides and that of their retro-counterparts [17]. Calculations performed using the hydrophobicity coefficient developed by Tripet B. et al. [18] suggests that the retro-analog of KR12-NH_2_, as well as its *N*-terminally acylated derivative, is more hydrophilic than the parent peptides (see Supplementary Materials, Table S2). The adjusted retention times of **II** and **XII** (6.71 vs. 5.64 min), as well as **III** and **XI** (5.24 vs. 5.05 min), confirmed the predictions. Peptides with branched carboxylic acids have lower retention times than those of peptides with straight-chain fatty acids of the same number of carbon atoms (**II** vs. **XIV**). This finding is in agreement with our previous results [14,15]. Usually, the number of carbon atoms in a lipophilic residue attached to a peptide is linearly correlated with its hydrophobicity (retention time) [12]. To verify this issue, the number of carbon atoms in the conjugated aliphatic acids is plotted against the adjusted retention time in Figure 1A–C.

In general, the hydrophobicity (retention time) of the KR12-NH_2_ analogs is moderately linearly correlated (R^2^ = 0.7502) with the number of carbon atoms in conjugated carboxylic acid residues (Figure 1A). KR12-NH_2_ and only its analogs with acylated *N^α^* or *N^ε^* or both amino groups of *N*-terminal lysine (**I**–**III**, **IX**, **X**, **XIII**, **XIV**) were analyzed (Figure 1B). This subgroup is more homogenous in terms of chemical structure. In conclusion, the coefficient of determination is higher (0.9216) when the similarity of the peptides increases. It can be seen that the analog with deoxycholic acid (**XXII**) differs distinctly from the others. In spite of the large number of carbon atoms (24), analog **XXII** has a relatively low hydrophobicity. This is due to its branched and polycyclic structure of deoxycholic acid and the presence of two hydrophilic hydroxyl groups.

Another group of analogs that can be distinguished is that containing aromatic carboxylic acids conjugated with the *N^α^*-amino group of KR12-NH_2_. The adjusted retention times of the analogs with aromatic carboxylic acid residues (**XV**–**XXI**) vs. the number of carbon atoms in the acid residue are shown in Figure 1C.

It was found that the hydrophobicity (adjusted retention time) and the number of carbon atoms of the carboxylic acid residues are linearly correlated (R^2^ = 0.8860). The correlation was even better after the exclusion of peptides **XIX** and **XX** (blue dots) (R^2^ = 0.9709). These two compounds contain carboxylic acids with double (**XIX**) and triple (**XX**) bonds between the phenyl and carbonyl entities. Together with analog **XVII,** they can be analyzed in terms of bond order/saturation (3-phenyl propionic acid, trans-cinnamic acid, and phenylpropiolic acid). According to the predicted [19] LogP values of 3-phenyl propionic acid, trans-cinnamic acid, and phenylpropiolic acid (and their amides), the hydrophobicity of the molecule increases with bond order. The estimated LogP is plotted against the bond order in Figure 1D.

The retention time of KR12-NH_2_ analogs with aromatic carboxylic acid residues with the same number of carbon atoms but a different bond order (peptides **XVII**, **XIX**, and **XX**; Figure 1E) did not follow the trend that could be deduced from the corresponding LogP values (Figure 1D, linear correlation). Seemingly, the hydrophobicity was not the only factor affecting the retention time. In this case, the acid residues had not only different bond orders and LogP, but also different shapes. The hybridization of carbon atoms has its consequences on molecule geometry. It has been shown that the shape of a molecule can significantly affect the retention behavior in HPLC [20].

### 2.2. Antibacterial Activity of the Peptides against ESKAPE Pathogens and S. aureus Strains

In our recent study, we examined the antibacterial activity of KR12-NH_2_ analogs with either n-alkyl acids (C_2_-C_14_) or aromatic acids (benzoic and trans-cinnamic) attached to the *N*-terminus. The experiments showed that almost all modifications improved the antibacterial activity of the compounds with regard to KR12-NH_2_. This is characteristic of most reference ESKAPE and *S. aureus* strains [12]. In view of the promising results, we decided to carry on research with KR12-NH_2_ analogs. In this paper, we tested conjugates with lipophilic molecules positioned at different sites of the KR12-NH_2_ molecule against ESKAPE pathogens (Table 2) and *S. aureus* strains (Table 3).

Among the ESKAPE strains most of the peptides showed higher antibacterial activity than that of KR12-NH_2_ (**I**), with peptides **II**, **XIII,** and **XXI** being the most active. In turn, the highest minimum inhibitory concentrations (MICs), still comparable to the activity of the parent KR12-NH_2_, were recorded for compounds **IX**, **X**, **XI,** and **XII.** These results indicate that the introduction of two fatty acid chains (**IX** and **X**) and the reversal of the amino sequence (**XI** and **XII**) turned out to be the least effective modifications of the KR12-NH_2_ peptide in the design of antibacterial compounds against the ESKAPE strains. The only exception was the activity against *E. faecium*. This strain was the most sensitive to all the synthesized analogs. All the MIC values against *E. faecium* were in the range of 2 to 32 µg/mL. Even the analogs with the lowest antibacterial activity against the remaining ESKAPE strains inhibited the growth of *E. faecium* at concentrations 4–15 times lower than that of KR12-NH_2_.

A similar tendency was maintained in the antibacterial assays with *S. aureus*. Peptides **II**, **XIII,** and **XXI** exhibited the highest activity against all the tested *S. aureus* strains. In turn, the highest MIC values were recorded for peptides **IX**, **X**, and **XI**, but the *S. aureus* ATCC 6538 strain still remained relatively sensitive (the MIC ranged between 16 and 64 µg/mL). It should be noted that the C_8_^α^-retro-KR12-NH_2_ peptide (**XII**) exhibited better antibacterial properties in assays with *S. aureus* strains than in tests with bacteria from the ESKAPE group (with the MIC ranging between 8 and 32 µg/mL vs. 8 and 256 µg/mL). This can be explained in terms of the differences in Gram-positive and Gram-negative bacteria (peptide **XII** was the most effective against Gram-positive microorganisms).

### 2.3. Hemolytic Activity and Its Relationship with Hydrophobicity

**Figure 2:**
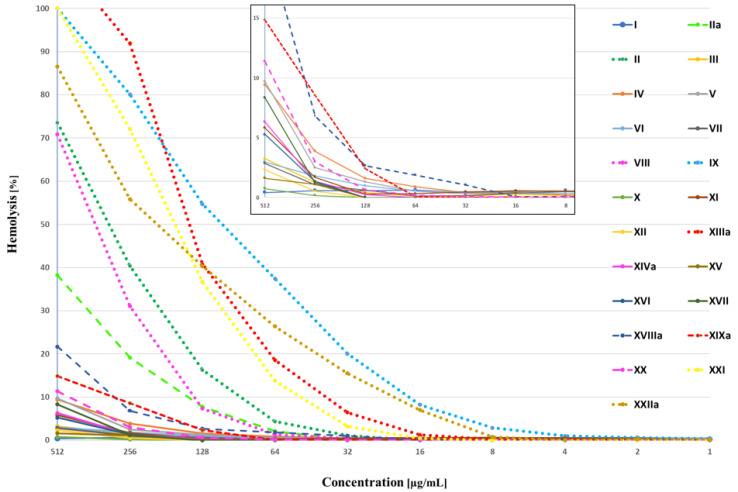
Percentage of hemolysis of erythrocytes vs. peptide concentration. ^a^ denotes fresh batch of blood to receive red blood cells.

The hemolytic activity of all the peptides against hRBCs is shown in Figure 2. To facilitate a comparison of hemolytic activity between the analogs, MHC_5_ values were determined. MHC_5_ is the minimum hemolytic concentration of the compounds that caused the 5% hemolysis of hRBCs. MHC_5_ values are presented in Table 4.

The majority of the studied analogs, except **VIII**, **IX**, **XIII**, **XXI,** and **XXII,** were less hemolytic than the parent compound (**II**). Only peptides **IX**, **XIII,** and **XXII** triggered hemolysis below their MIC values.

**Figure 3:**
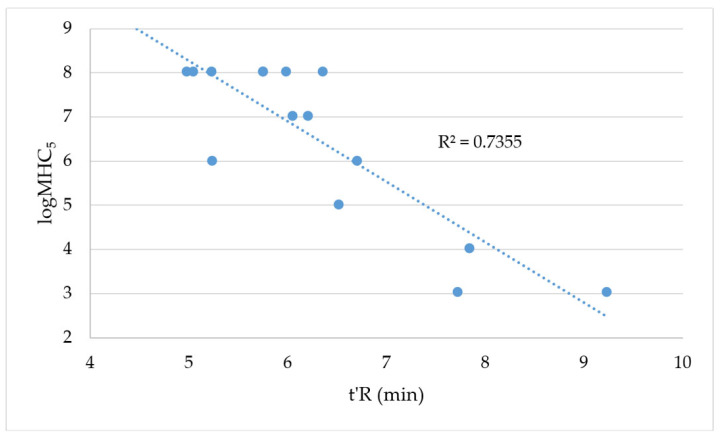
Log_2_MHC_5_ vs. t’R.

Usually, the hemolytic activity of membrane-active compounds is correlated with their hydrophobicity. As has been reported, the lysis of red blood cells increases with an increase in the lipopeptide hydrophobicity (the length of the acyl chain) [6,12,14,21]. The logMHC_5_ vs. the adjusted retention time is plotted in Figure 3.

It can be seen that the hemolytic activity of the lipopeptides used in this study was linearly correlated with their adjusted retention time (R^2^ = 0.7355). However, it is rather difficult to discuss the influence of the lipopeptide charge on hemolysis due to the relatively small pool of compounds of charges +3 and +5. It can only be stated that the most hydrophobic compounds were the most hemolytic, i.e., **IX, XIII,** and **XXII**. A few compounds (seven) did not exhibit a hemolytic effect over the concentration range studied (up to 512 μg/mL) and their t’R was between 3.81 and 6.29 min. This range overlaps with the hydrophobicity of these compounds that caused substantial hemolysis; hence, it can be concluded that the position of the substitution/modification (the conjugation of carboxylic acid) can play a significant role in membrane lysis. The modification of different amino acid residues in the helical peptide (KR12-NH_2_) can produce molecules of various hydrophobic moments that are important in peptide–membrane interactions [22].

### 2.4. Selectivity Index: S. aureus Strains

Selectivity indexes were calculated based on the MHC_5_ and geometric mean (GM) of MIC values against *S. aureus* strains. The results are presented in Table 5.

The switching of the lipidation site from an α-amino group of the *N*-terminal lysine residue of peptide KR12-NH_2_ to its side chain resulted in a significant increase in MHC_5_ from 64 (peptide **II**) to 512 µg/mL (peptide **III**). A similar approach to conjugates with an additional Lys residue attached to the *N*-terminus of KR12-NH_2_ (peptides **IV** and **V**) did not differentiate the compounds in respect to their hemolytic properties. However, these compounds were less toxic than peptide **II**. Overall, extending the KR12-NH_2_ sequence with an additional Lys residue at the *N*-terminus, together with changing the lipidation site from the α-amino group to the ε-amino group of the *N*-terminal lysine, reduced the hydrophobicity of the peptides, as reflected by HPLC retention times. The transfer of the additional side chain-lipidated Lys from the *N*-terminus (**V**) to the *C*-terminus of KR12-NH_2_ (**VI**) resulted in a further drop in the hemolysis of hRBCs (the MHC_5_ was 256 µg/mL and 512 µg/mL, respectively), with this complying with the findings by Albada et al. [13,24]. A similar effect was noticed for a counterpart with a *C*-terminal arginine residue replaced by side chain-lipidated lysine residue (**VII**). However, peptide **VII** had a six-times-lower selectivity index than peptide **VI** (Table 5). In turn, the conjugation of the octanoic acid to the side chain amino group of Lys at position 8 in the KR12-NH_2_ molecule produced peptide **VIII,** as toxic as compound **II** and with an approximately five-fold-lower selectivity index (SI).

In the next step, we decided to check how the lipidation site would affect the activity of the retro-KR12-NH_2_ analogs (peptides **XII** and **XI**) as compared to the parent counterparts (**II** and **VI**). Our results show that the *N*-terminus of the retro-KR12-NH_2_ was a more favorable lipidation site. Peptide **XII** had a much higher selectivity index (>21) than that of peptide **XI** (1.52) and a slightly higher one than peptides **II** and **VI** (18.39 and 12.13, respectively) in the case of *S. aureus* strains. In the literature, no information could be found on the lipidation of retro-analogs. We only know from our earlier experiments that, usually, retro-peptides show decreased antibacterial and hemolytic activity as compared to that of the parent molecule (aurein 1.2, CAMEL, citropin 1.1, pexiganan, and temporin A). However, in the case of omiganan and its retro-analog, an opposite effect has been reported [17].

The next-designed peptide **IX** containing two octanoic acid residues in the *N*-terminal part of the KR12-NH_2_ molecule exhibited significant hemolytic activity and the lowest selectivity index of 0.04 (Table 5). While the attachment of two shorter acyl chains (butanoic acid residues) to the lysine residue at position 1 in the KR12-NH_2_ molecule (**X**) produced a less hydrophobic compound than analogs **IX** and **II**, which did not induce any significant hemolysis (>5%) over the studied concentration range (0.5–512 µg/mL), at the same time, its antibacterial activity dropped significantly (the MICs were between 32 and 512 µg/mL); consequently, its selectivity index was seven times lower than that of peptide **II**. Greber et al. showed that for the double-chain multi-lysine lipopeptides: (C_8_)_2_-KKKK-NH_2_, (C_10_)_2_-KKKK-NH_2_, (C_12_)_2_-KKKK-NH_2_, (C_14_)_2_-KKKK-NH_2_, and (C_16_)_2_-KKKK-NH_2_, the analog with two octanoyl residues was the least toxic, with no significant hemolytic activity monitored over the whole concentration range tested (up to 512 µg/mL), in contrast to our peptide **IX** [25].

The substitution of hydrogen at position 2 of the octanoic acid residue in peptide **II** with a butyl group resulted in peptide **XIII** with a significantly increased toxicity (MHC_5_ = 8 µg/mL) and a seven-times-decreased SI value as compared to that of the parent peptide. A more favorable modification appeared to be coupling a branched isomer of octanoic acid (2-ethylhexanoic acid) with the KR12-NH_2_ molecule that gave analog **XIV** lower hemolytic activity than that of peptide **II,** while its antibacterial activity slightly dropped and, consequently, the selectivity index was reduced twice.

In analogs **XV**–**XVIII** modified with C_6_H_5_-X-COOH (where X: CH_2_, CH_2_-CH_2_, and CH_2_-CH_2_-CH_2_), we studied the impact of the distance between the phenyl ring and the carboxylic group on their toxicity. The hemolytic activity of the peptides gradually increased together with the lengthening of the spacer in the acid molecule and with an increase in the hydrophobicity of the peptides. However, these results did not correlate with their selectivity. In turn, an increase in the multiplicity of the C^2^-C^3^ bond in the hydrocarbon linker in the analog **XVII** resulted in the counterparts **XIX** and **XX** (with a double and a triple bond, respectively) which did not affect their hemolytic activity. The SI indexes of peptides **XVII**, **XIX,** and **XX** dropped with an increasing multiplicity of the C^2^-C^3^ bond. The attachment of an additional phenyl ring to analog **XV** led to peptide **XXI** that exhibited significantly higher antibacterial and hemolytic activity than the parent compound. It is worth noting that the antibacterial activity of peptide **XXI** was comparable to that of peptide **II** and was only slightly more toxic than that of the led compound with its selectivity index against *S. aureus* strains dropping twice in comparison to the parent compound. An analog modified with deoxycholic acid (**XXII**) exhibited significant hemolytic activity (MHC_5_ = 8 µg/mL) and was as hemolytic as compound **IX** which was much more hydrophobic.

**Figure 4:**
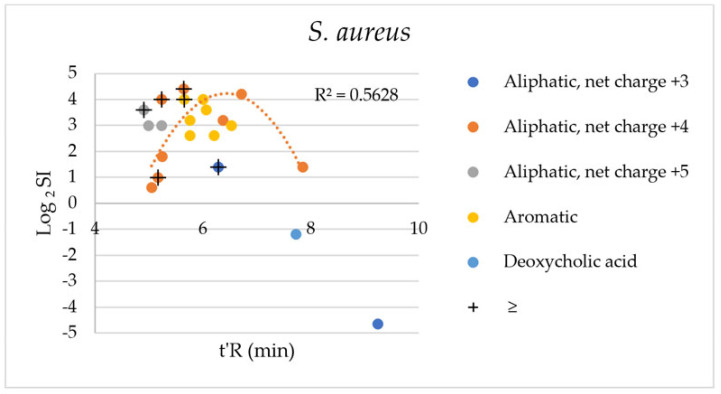
Log_2_SI vs. t’R-*S. aureus*. + refers to SI values equal or higher than the calculated one (MHC_5_ ≥ 512 µg/mL).

Biological activity, both antimicrobial and hemolytic, can be associated with peptide hydrophobicity. The selectivity index calculated for *S. aureus* strains (log_2_SI) vs. the adjusted retention time (t’R) is plotted in Figure 4.

It can be seen that most of the peptides have a higher selectivity to *S. aureus* cells than erythrocytes. The only exceptions are compounds **IX** and **XXII**, which are highly hydrophobic. Interestingly, peptide **XIII** with a 2-butyloctanoic acid residue has a substantially higher SI despite the similar retention time. The modification of KR12-NH_2_ with deoxycholic acid led to an analog with high hemolytic potency. It was found that the cell membrane of human erythrocytes contains equal proportions by weight of cholesterol and phospholipids which are equally distributed between the two leaflets [26]. Moreover, deoxycholic acid, being an analog of cholesterol, can effectively interact with it. Interactions with membranes can be partially associated with the intrinsic amphipaticity of the deoxycholic acid residue. It was shown that deoxycholic acid can promote the aggregation and redistribution of cholesterol in the membrane (HCT116 cells) and promote membrane perturbations [27,28]. There is a moderate quadratic correlation between the Log_2_SI of the analogs with aliphatic carboxylic acid residues of net charge +4 and their t’R (R^2^ = 0.5628). This trend indicates that between the distinctive hydrophobic and hydrophilic compounds of low SI are those with optimal lipophilicity and the highest selectivity. Indeed, analogs **II** and **XII** of unremarkable hydrophobicity are the most selective ones. Similar patterns between the selectivity over human cells and hydrophobicity were found for ultrashort cationic lipopeptides [14].

### 2.5. Selectivity Index: ESKAPE Strains

Selectivity indexes were calculated based on MHC_5_ and MIC values against *A. baumannii*, *P. aeruginosa*, *K. aerogenes*, *K. pneumoniae*, and *E. faecium* strains. The results are presented in Table 6.

Our previous studies indicate that the most beneficial acyl chain length is at the *N*-terminus of KR12-NH_2_ is C_8_ (octanoic acid, compound **II**). The selectivity indexes which are higher than that of peptide **II** are in bold (Table 6). There is no analog more selective against *K. aerogenes* over RBCs than **II**, but there are two peptides with equal SI values (**XIV** and **XX**). Nonetheless, three peptides have higher SIs in the case of all remaining bacteria strains than that of compound **II**, namely analogs **III**, **IV**, and **VI**. The remaining three compounds are more effective in terms of selectivity against three out of five strains, i.e., peptides **V**, **VII**, and **XIV**. Interestingly, analogs **IV**, **V**, and **VI** have the highest net charge (+5) over those of the synthesized peptides. This finding highlights the crucial role of peptide charge in biological activity, selectivity and, thus, in therapeutic potential. Both peptides **III** and **VII** have octanoic acid conjugated to the *N^ε^*-amino group of lysine. However, peptide **VIII** with the same type of modification as **III** and **VII** and net charge (+4) is much less selective. Analogs **III** and **VII** have a modified lysine residue at the terminal positions (*N-* and *C*-terminal, respectively) but **VIII** has this modification between those ends at eight amino acid residues. This once again emphasizes the importance of the position of amino acid residues modified by lipidation. The hydrophobicities of **III**, **VII**, and **VIII** are comparable but their SIs are radically different. Moreover, branched carboxylic acid seems to be more beneficial over the straight-chain one with an identical number of carbon atoms to peptide selectivity (**XIV** vs. **II**). This finding is in agreement with previous studies on short cationic lipopeptides and gemini lipopeptides which revealed an increased selectivity in compounds with branched acid residues [14,15].

### 2.6. Conformational Studies of the Peptides Using Circular Dichroism and the Relationship between Hydrophobicity and Helicity

**Figure 5:**
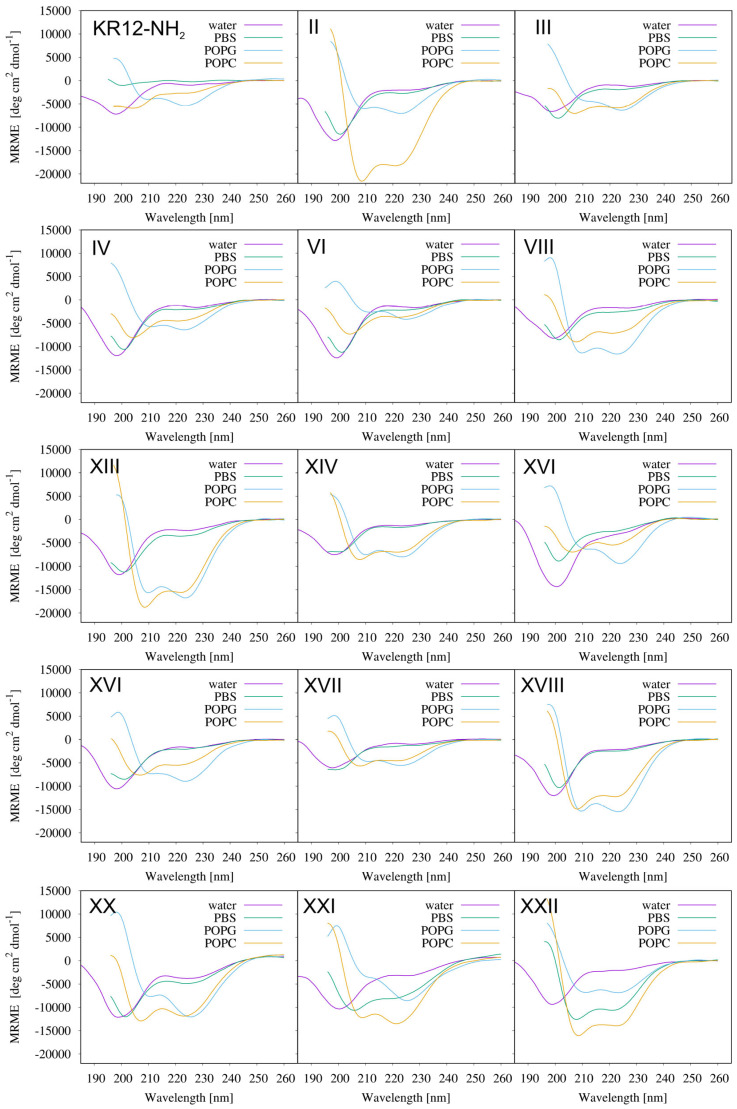
Far-UV CD spectra of the selected peptides recorded in water, PBS, POPG, and POPC solutions.

Far-UV CD spectra were recorded for the selected peptides under different environmental conditions (Table 7, Figure 5).

In the lipid-free solutions, the peptides assumed an unordered structure, except compounds **XX**, **XXI,** and **XXII** in PBS (Figure 5). These peptides tended to form an α-helix, as highlighted by the appearance of two characteristic minima at 208 and 222 nm in the CD spectra. This phenomenon was related to a higher ionic strength in the PBS solution compared to the aqueous solution, which was accompanied by an increased tendency of the peptides to self-assembly. In PBS, the high salt concentration reduced the electrostatic repulsion between the peptide monomers by screening the positive charges on the amino acid side chains and facilitated the self-assembly. In the case of peptides **XX** and **XXI**, the higher tendency to self-assemble can be also attributed to the presence of the additional aromatic ring(s) and π-π stacking interactions [29,30]. However, taking into account that not all of the aromatic counterparts showed a tendency to self-assemble at a concentration of 0.15 mg/mL, it can be assumed that the increase in aromatic acid rigidity was additionally conducive to self-assembly. In turn, compound **XXII** combined the aggregation properties of deoxycholic acid [31] and amphipathic α-helical peptides. The amphiphilicity and high structural rigidity of deoxycholic acid are known to promote self-association in solution, which is driven by both hydrophobic association and H-bonding interactions [32].

**Figure 6:**
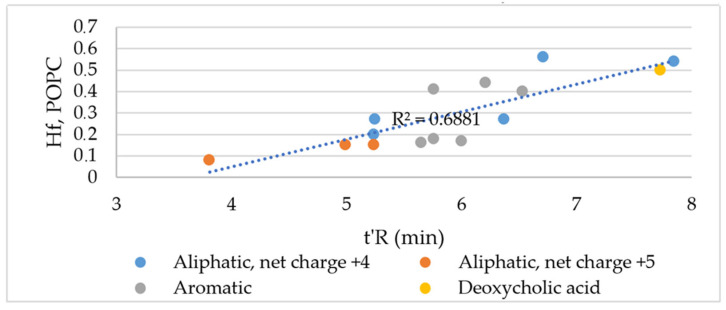
Helical fraction (Hf) vs. adjusted retention time.

All the studied compounds folded into an α-helical structure after the transfer from an aqueous into POPG (palmitoyl-2-oleoyl-sn-glycero-3-phosphoglycerol) and POPC (1-palmitoyl-2-oleoyl-glycero-3-phosphocholine) solutions. These results indicated interactions between the peptides and both artificial membranes (Table 7). Interestingly, the Ө_222_/Ө_208_ > 1 noticed for all the studied compounds indicated a coiled-coil structure and, thus, peptide aggregation in the presence of negatively charged POPG vesicles. Additionally, in the case of peptides **XX** and **XXI**, the 222 nm minimum was red-shifted toward 224 nm, similarly to the peptides forming coiled-coil fibers [32,33]. In contrast to KR12-NH_2_, the peptides also adopted a helical structure in the presence of zwitterionic POPC liposomes. The helical fraction (Hf) of the peptides in different media vs. the adjusted retention time is plotted in Figure 6 (POPC) and Figure S1 (other media, Supplementary Materials).

In general, there was no linear correlation between the hydrophobicity and helicity of the studied peptides in the negatively charged artificial POPG membrane (Figure S1, see Supplementary Materials). In turn, the correlation coefficient (R^2^ = 0.6881) found for the peptides in POPC, which mimics eukaryotic membranes, suggested only a moderate linear correlation (Figure 6). The peptides **II**, **XIII**, **XVIII**, **XX**, **XXI,** and **XXII** with the highest tendency to form the α-helical structure in POPC exhibited significant hemolytic activity and that was associated with an increase in their hydrophobicity.

### 2.7. Effects of the Peptides on Liposome Aggregation

**Figure 7:**
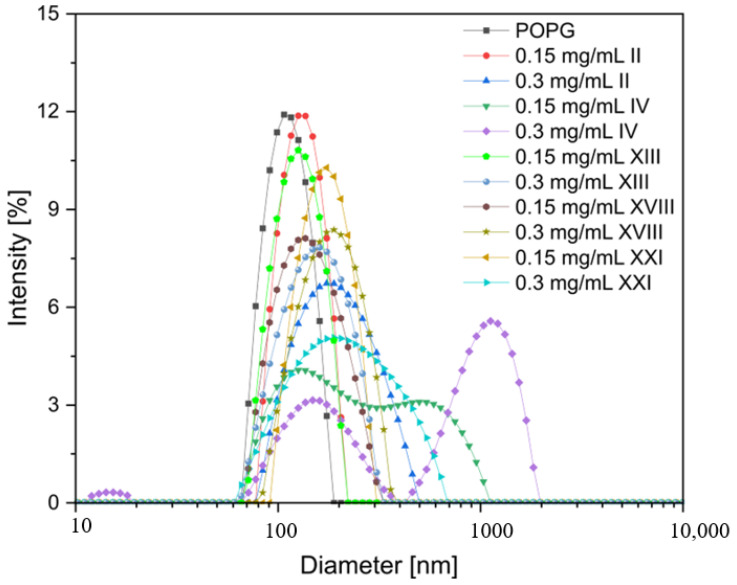
Dynamic light scattering distribution curves of the POPG LUVs’ hydrodynamic diameter in the absence and presence of selected peptides.

Dynamic light scattering (DLS) measurements were performed to determine the POPG LUVs’ size variation induced by peptide conjugates. Here, identical concentrations of the peptides and lipids as in the CD experiment (0.15 mg/mL and 1.3 mM, respectively) were used. Additionally, the DLS measurements were repeated with a twice as high peptide concentration (0.3 mg/mL). The results showed that the peptides promoted the aggregation of the negatively charged large unilamellar POPG vesicles (LUVs) (Table 8). In a peptide-free solution, the POPG LUVs had a size distribution centered at 113.8 ± 24.6 nm. Upon the addition of the peptide conjugates, the hydrodynamic diameter (D_H_) of the POPG LUV increased, with the effect being more pronounced for a higher peptide concentration (Figure 7). In the case of peptides **II** and **XXI** at a concentration of 0.3 mg/mL, an approximately two-fold increase in the D_H_ of POPG LUVs was noticed. In turn, with the POPG LUVs in the presence of peptide **IV**, the polydisperse particle size distribution profiles were detected with the onset of two independent populations. These larger entities corresponded to the aggregation or fusion of the liposomes induced by the peptides. The variations in the POPG liposomes’ size were not accompanied by those in zeta potential over the studied peptide concentration range. The zeta potential, as an indicator for accessible surface charges, determined for the pure POPG was −36.7 ± 1.7 mV. The addition of the peptides at the concentrations of 0.15 and 0.3 mg/mL induced only a slight zeta potential variation, mostly within the standard deviation range. The largest variation was found for the POPG/IV complex, which was in good agreement with the highest overall positive charge of peptide **IV** among the studied compounds. However, it is hypothesized that liposome aggregation was promoted by both the partial neutralization of the vesicle surface and the interhelical interactions between peptide molecules anchored in the adjacent POPG vesicles. The contribution of the interhelical interaction in liposome aggregation is even more likely as the CD spectra of the peptides in the POPG liposomes clearly showed the presence of coiled-coil structures.

## 3. Materials and Methods

### 3.1. Peptide Synthesis and Purification

All the peptides (Table 1) were synthesized with the solid-phase method using Fmoc chemistry on a resin modified by a Rink amide linker with a loading of 1.0 mmol/g (Orpegen Peptide Chemicals GmbH, Heidelberg, Germany) [34]. *N*-Fmoc-protected amino acids and the coupling reagents were obtained from Iris Biotech GmbH (Marktredwitz, Germany); we also obtained trifluoroacetic acid (TFA) (Apollo Scientific, Denton, UK) and piperidine (Iris Biotech GmbH, Marktredwitz, Germany). The solvents and other reagents were purchased from Sigma-Aldrich (Poznan, Poland). The following amino acid derivatives were used: Fmoc-Gln(Trt)-OH, Fmoc-Asp(OtBu)-OH, Fmoc-Lys(Boc)-OH, and Fmoc-Arg(Pbf)-OH. In addition, in some analogs, Boc-Lys(Fmoc)-OH (**III**, **V**), Fmoc-Lys(Fmoc)-OH (**IX**, **X**), and Fmoc-Lys(C_8_)-OH (**VI**, **VII**, **VIII**, **XI**) were also used.

The synthesis of peptides was carried out on a microwave peptide synthesizer Biotage Initiator+Alstra (Shim-pol, Warszawa, Poland). The peptide chains were elongated in consecutive cycles of deprotection and coupling. The deprotection of the Fmoc group was performed twice in a 20% (*v*/*v*) piperidine solution in *N*,*N*-dimethylformamide (DMF) for 3 and 10 min. The acylation of all protected amino acids, except for the Arg derivative, was conducted twice (2 × 5 min) at 75 °C in a DMF solution with coupling agents using a 3-fold molar excess of *N*,*N*-diisopropylcarbodiimide (DIC) and OxymaPure. The coupling of Fmoc-Arg(Pbf)-OH was carried out twice at RT for 1 h. After the synthesis was completed, the peptide resin was dried. The coupling reactions of lipophilic residues (fatty acids and aromatic acids) with the *N*-terminal part of molecules were performed using the same method as that used for the protected arginine.

All the peptides were removed from the resin along with the side chain deprotection in a one-step procedure using a mixture of TFA, triisopropylsilane (TIS), and water (95:2.5:2.5 *v*/*v*/*v*) for 2 h. Then, the peptides were precipitated with cold ether diethyl and lyophilized.

Finally, the peptides were purified with solid-phase extraction (SPE) on Isolute ^TM^ SPE columns (flash, C_18_, 25 mL) [35]. The eluates were fractionated, and the purity of the peptides was determined on a Varian ProStar HPLC system controlled by a Galaxie Chromatography Data System with a Phenomenex Gemini-NX C18 column (4.6 × 150 mm, 110 Å pore size, 5 µm particle size). The solvent systems used were: 0.1% aqueous TFA (A) and 0.1% TFA in acetonitrile (ACN) (B). UV detection at 214 nm was used, and the peptides were eluted with a linear gradient 10–100% B in A over 10 min at 25 °C. The mobile-phase flow rate was 0.5 mL/min.

The ESI MS (Waters Alliance e2695 system with Acquity QDA detector, Waters, Milford, MA, USA) was used to determine the masses of the peptides.

### 3.2. Antibacterial Assays

Tests of the antibacterial activity were carried out as we just described. Briefly, for assays, the following strains of ESKAPE bacteria were chosen: *Acinetobacter baumannii* ATCC BAA-1605, *Enterococcus faecium* ATCC 700221, *Klebsiella aerogenes* ATCC 13048 (formerly *Enterobacter aerogenes*), *Klebsiella pneumoniae* ATCC 700603, and *Pseudomonas aeruginosa* ATCC 9027. Moreover, the reference strains of *Staphylococcus aureus* were used: *S. aureus* ATCC 25923, *S. aureus* ATCC 6538, *S. aureus* ATCC 33591 (MRSA), *S. aureus* ATCC 9144, and *S. aureus* ATCC 12598. Before experiments, all the strains were stored at −80 °C in Roti^®^-Store cryo vials (Carl Roth GmbH, Karlsruhe, Germany). To evaluate the activity of the compounds, bacteria were transferred into fresh Mueller–Hinton medium (Becton Dickinson, Warszawa, Poland) and incubated for 24 h at 37 °C. In the next step, bacterial inoculums were seeded on Mueller–Hinton agar plates (BioMaxima SA, Lublin, Poland) and incubated again for 24 h. The MIC values were determined using the broth microdilution method according to the Clinical and Laboratory Standards Institute Protocol [36]. The initial inoculums of bacteria (0.5 × 10^5^ colony forming units (CFU)/mL) prepared in Mueller–Hinton broth were exposed to the ranging concentrations of peptides (1–512 µg/mL). The experiments were conducted on 96-well microtiter plates, with a final volume of 100 µL. The inoculum densities were checked spectrophotometrically (Multiskan™ GO Microplate Spectrophotometer, Thermo Scientific, Waltham, MA, USA) at 600 nm. The results were read after incubation for 18 h at 37 °C. The MIC values were the lowest concentration at which a visible growth of microorganisms was inhibited [37]. All experiments were conducted in triplicate. Moreover, positive (growth) and negative (sterility) controls were included.

### 3.3. Hemolysis Assay

The assay was conducted according to the procedure reported by Avrahami and Shai [38]. Briefly, the fresh hRBCs with EDTA as anticoagulant were rinsed three times with PBS through centrifugation at 800× *g* for 10 min and resuspended in PBS. The serial dilution of peptides (1–512 µg/mL) was prepared in PBS on 96-well plates. Then, the stock RBCs solution was added to the plates to reach a final volume of 100 µL with a 4% concentration of erythrocytes (*v*/*v*). The control wells for 0 (RBCs in PBS) and 100% (RBCs in 1% Triton-X 100) hemolysis were also prepared. Then, the plates were incubated for 1 h at 37 °C and centrifuged at 800× *g* for 10 min at 4 °C (Sorvall ST 16R Centrifuge, Thermo Scientific, Waltham, MA, USA). After centrifugation, the supernatant was carefully transferred to new microtiter plates and the release of hemoglobin was measured at 540 nm (Multiskan™ GO Microplate Spectrophotometer, Thermo Scientific, Waltham, MA, USA). The percentage of hemolysis was calculated based on wells with 100% hemolysis.

### 3.4. CD Measurements

Circular dichroism studies were performed at 25 °C in water, 10 mM PBS buffer (pH 7.4), and POPG and POPC large unilamellar vesicles (LUVs), with a total lipid concentration of 1.3 mM. Large unilamellar vesicles (LUVs) ~100 nm in diameter were prepared using extrusion according to the previously described procedure [39]. The CD spectra were recorded over the range of 185–260 nm with a JASCO-815 spectropolarimeter in a 1 mm pathlength cuvette and a peptide concentration of 0.15 mg/mL. The spectra were corrected by subtracting the background from the sample spectrum and plotted as mean residue molar ellipticity (degree × cm^2^ × dmol^−1^) vs. wavelength (nm). The measurements were repeated three times to increase the signal-to-noise ratio. The content of the helical structure was calculated from the spectra using the CONTINLL method implemented in the CDPro package [40].

### 3.5. Dynamic Light Scattering Measurements

Dynamic light scattering experiments were carried out on a Litesizer 500 (Anton-Paar GmbH, Graz, Austria) at 25 °C with a backscattering detection (175°) using disposable polystyrene cuvettes. The POGP LUV suspensions were diluted to the desired final concentration of 1.3 mM. The peptide concentrations were kept at 0.15 and 0.3 mg/mL (~0.1 and 0.2 mM, respectively). The hydrodynamic diameters were obtained from the peaks with the highest scattered-light intensity in the light-scattering intensity distribution.

The zeta potential of the liposomes was determined at 25 °C in the absence and presence of the selected peptides at two different concentrations (0.15 and 0.3 mg/mL), via electrophoretic light scattering in the above-mentioned apparatus, using an omega-shaped zeta potential cell. The viscosity value and refractive index were set at 0.904 mPa·s and 1.33, respectively.

## 4. Conclusions

Lipidation is one of the valuable methods used to increase the antimicrobial activity of AMPs. However, there are only scarce reports on the effect of the degree and position of the fatty acid chains in lipoAMPs, which potentially could affect the structural arrangement of the peptides and the mode of interaction with bacterial and eukaryotic cell membranes, and, consequently, modulate their selectivity against them. The results of this work can be summarized:

- Most of C_8_^α^-KR12-NH_2_ (**II**) analogs that were more hydrophobic than the parent compound were also more hemolytic. A similar relationship was also found between the α-helical structure content in POPC and hemolytic activity. The only exceptions were compounds **VIII** and **XXI**, which exhibited comparable or higher toxicity, respectively, than peptide **II** despite lower hydrophobicity.
- All lipidated analogs with the highest net charge (+5) were the most selective toward pathogens (analogs **IV**, **V** and **VI**); therefore, the overall charge of KR12-NH_2_ analogs plays a pivotal role in their biological activity and must be considered in the further optimization of therapeutic potential.
- The conjugation of the branched carboxylic acid (2-ethylhexanoic acid, **XIV**) to the *N*-terminus of the KR12-NH_2_ molecule and the transfer of the lipophilic moiety from the *C*-terminal lysine side chain to the *N*-terminal lysine (**VII** and **III**, respectively) or vice versa for analogs with the additional lysine (**V** and **VI**, respectively) are preferred tactics to increase peptide selectivity.
- It is worth emphasizing that in our study, the highest selectivity against *S. aureus* strains with an SI value of at least 21.11 exhibited peptide **XII** obtained using the conjugation of the octanoic acid with the *N*-terminus of retro-KR12-NH_2_.
- The increase in the multiplicity of the C^2^-C^3^ bond in the hydrocarbon linker (analogs **XVII**, **XIX**, and **XX**) correlates with the decrease in SI values against *S. aureus* strains.
- The increase in the degree of lipidation of the peptide KR12-NH_2_ significantly reduced its antimicrobial activity against both ESKAPE and *S. aureus* strains (analog **IX**) and SI values.
- We showed that the representative lipoAMPs that exhibited high antibacterial activity promoted the aggregation of the negatively charged large unilamellar POPG vesicles (LUVs).

Our study proved that the lipidation of KR12-NH_2_ can result in peptides with improved selectivity against bacterial pathogens over human cells. It seems that selectivity can be elevated with increasing net charge and branched carboxylic acids. the Results confirmed that the position of the lipidated amino acid residue in KR12-NH_2_ is crucial for biological activity.

## Figures and Tables

**Table 1:** Characteristics of the peptides.

No.	Peptide	Sequence	Net Charge	No. of Carbons Atoms in Lipophilic Entity	HPLC t_R_ (min)
I	KR12-NH_2_	KRIVQRIKDFLR-NH_2_	+5	0	3.81
II	C_8_^α^-KR12-NH_2_	C_8_-KRIVQRIKDFLR-NH_2_	+4	8	6.71
III	C_8_^ε^-KR12-NH_2_	K(C_8_)RIVQRIKDFLR-NH_2_	+4	8	5.24
IV	C_8_^α^-Lys-KR12-NH_2_	C_8_-KKRIVQRIKDFLR-NH_2_	+5	8	5.24
V	C_8_^ε^-Lys-KR12-NH_2_	K(C_8_)KRIVQRIKDFLR-NH_2_	+5	8	4.99
VI	KR12-Lys^ε^(C_8_)-NH_2_	KRIVQRIKDFLRK(C_8_)-NH_2_	+5	8	4.91
VII	[Lys^ε^(C_8_)]^12^KR12-NH_2_	KRIVQRIKDFLK(C_8_)-NH_2_	+4	8	5.17
VIII	[Lys^ε^(C_8_)]^8^KR12-NH_2_	KRIVQRIK(C_8_)DFLR-NH_2_	+4	8	5.25
IX	C_8_^α^,C_8_^ε^-KR12-NH_2_	C_8_-K(C_8_)RIVQRIKDFLR-NH_2_	+3	16	9.24
X	C_4_^α^, C_4_^ε^-KR12-NH_2_	C_4_-K(C_4_)RIVQRIKDFLR-NH_2_	+3	8	6.29
XI	retro-KR12-C_8_^ε^-NH_2_	RLFDKIRQVIRK(C_8_)-NH_2_	+4	8	5.05
XII	C_8_^α^-retro-KR12-NH_2_	C_8_-RLFDKIRQVIRK-NH_2_	+4	8	5.64
XIII	2-Buthyloctanoic acid-KR12-NH_2_	CH_3_-(CH_2_)_5_-CH(C_4_H_9_)-CO-KR12-NH_2_	+4	12	7.85
XIV	2-Ethylhexanoic acid-KR12-NH_2_	CH_3_-(CH_2_)_3_-CH(C_2_H_5_)-CO-KR12-NH_2_	+4	8	6.37
XV	Benzoic acid-KR12-NH_2_	C_6_H_5_-CO-KR12-NH_2_	+4	7	5.65
XVI	Phenylacetic acid-KR12-NH_2_	C_6_H_5_-CH_2_-CO-KR12-NH_2_	+4	8	5.76
XVII	3-Phenylpropionic acid- KR12-NH_2_	C_6_H_5_-CH_2_-CH_2_-CO-KR12-NH_2_	+4	9	6
XVIII	4-Phenylbutanoic acid-KR12-NH_2_	C_6_H_5_-(CH_2_)_3_-CO-KR12-NH_2_	+4	10	6.21
XIX	trans-cinnamic acid-KR12-NH_2_	C_6_H_5_-CH=CH-CO-KR12-NH_2_	+4	9	6.06
XX	Phenylpropiolic acid-KR12-NH_2_	C_6_H_5_-C≡C-CO-KR12-NH_2_	+4	9	5.76
XXI	4-Phenylbenzoic acid- KR12-NH_2_	C_6_H_5_-C_6_H_4_-CO-KR12-NH_2_	+4	13	6.53
XXII	Deoxycholic acid- KR12-NH_2_	C_24_H_39_O_3_-KR12-NH_2_	+4	24	7.73

**Table 2:** The minimum inhibitory concentrations (MICs, µg/mL) of the peptides against reference strains of ESKAPE pathogens.

No.			*G*(−)		*G*(+)
	*A. baumannii*	*P. aeruginosa*	*K. aerogenes*	*K. pneumoniae*	*E. faecium*
I	256	128	>512	>512	128
II	8	16	4	8	4
III	32	64	64	32	4
IV	8	32	32	16	4
V	32	16	64	16	4
VI	16	32	64	32	4
VII	32	64	64	128	16
VIII	8	64	32	32	8
IX	512	>512	512	>512	16
X	128	>512	256	512	32
XI	64	128	512	512	32
XII	64	64	256	128	8
XIII	4	32	8	8	2
XIV	16	128	16	16	4
XV	32	128	64	64	8
XVI	32	128	64	64	8
XVII	32	64	64	32	4
XVIII	4	64	32	16	8
XIX	8	32	16	16	4
XX	32	64	16	32	4
XXI	8	16	4	8	2
XXII	8	128	128	64	2

**Table 3:** The MIC values (µg/mL) of the test peptides against reference strains of *Staphylococcus aureus*.

No.	*S. aureus 25923*	*S. aureus* *6538*	*S. aureus 33591*	*S. aureus* *9144*	*S. aureus* *12598*
I	>512	512	>512	>512	>512
II	4	2	4	4	4
III	32	8	64	32	64
IV	128	8	64	16	32
V	64	8	64	32	32
VI	64	64	32	32	32
VII	128	64	512	512	512
VIII	32	4	32	16	32
IX	512	64	256	256	256
X	128	32	512	256	512
XI	128	16	512	256	512
XII	32	8	32	32	32
XIII	8	32	4	2	4
XIV	64	16	16	32	32
XV	64	8	64	32	32
XVI	128	16	64	32	32
XVII	16	8	32	16	16
XVIII	32	32	16	8	32
XIX	16	16	8	4	16
XX	128	8	32	16	32
XXI	8	2	4	4	4
XXII	32	4	256	8	8

**Table 4:** MHC_5_ of the peptides.

No.	MHC_5_ (µg/mL)	No.	MHC_5_ (µg/mL)	No.	MHC_5_ (µg/mL)	No.	MHC_5_ (µg/mL)
I	≥512	VII	≥512	XIII	16 ^a^	XIX	128 ^a^
II	64	VIII	64	XIV	256 ^a^	XX	256
III	≥512	IX	8	XV	≥512	XXI	32
IV	256	X	≥512	XVI	256	XXII	8 ^a^
V	256	XI	256	XVII	256		
VI	≥512	XII	≥512	XVIII	128 ^a^		

^a^ denotes a fresh batch of blood to receive red blood cells. ≥means that investigated analog caused less than 5% hemolysis of human red blood cells over the whole tested concentration range (up to 512 µg/mL).

**Table 5:** GM ^1^ and selectivity index (SI ^2^) of peptides determined for reference strains of *S. aureus*.

No.	GM ^1^	SI ^2^ (MHC_5_/GM)	No.	GM ^1^	SI ^2^ (MHC_5_/GM)
I	>512	NA	XII	24.25	≥21.11
II	3.48	18.39	XIII	6.06	2.64
III	32	≥16	XIV	27.86	9.19
IV	32	8	XV	32	≥16
V	32	8	XVI	42.22	6.06
VI	42.22	≥12.13	XVII	16	16
VII	256	≥2	XVIII	21.11	6.06
VIII	18.38	3.48	XIX	10.56	12.12
IX	222.86	0.04	XX	27.86	9.19
X	194.01	≥2.64	XXI	4	8
XI	168.9	1.52	XXII	18.38	0.44

^1^ The geometric mean (GM) of the MIC values against *S. aureus* was calculated. ^2^ SI is the MHC/GM ratio. More selective compounds are characterized by the highest values of SI [23].

**Table 6:** Selectivity index determined for MIC against ESKAPE strains and MHC_5_.

No.			*G*(−)		*G*(+)
	*A. baumannii*	*P. aeruginosa*	*K. aerogenes*	*K. pneumoniae*	*E. faecium*
I	2	4	1	1	4
II	8	4	16	8	16
III	**16**	**8**	8	**16**	**128**
IV	**32**	**8**	8	**16**	**64**
V	8	**16**	4	**16**	**64**
VI	**32**	**16**	8	**16**	**128**
VII	**16**	**8**	8	4	**32**
VIII	8	1	2	2	8
IX	0.0156	0.0156	0.0156	0.0156	0.5
X	4	1	2	1	16
XI	4	2	0.5	0.5	8
XII	8	**8**	2	4	**64**
XIII	4	0.5	2	2	8
XIV	**16**	2	16	**16**	**64**
XV	**16**	4	8	8	**64**
XVI	8	2	4	4	**32**
XVII	8	4	4	8	**64**
XVIII	**32**	2	4	8	16
XIX	**16**	4	8	8	**32**
XX	8	4	16	8	**64**
XXI	4	2	8	4	16
XXII	1	0.0625	0.0625	0.125	4

The selectivity indexes which are higher than that of peptide **II** are in bold.

**Table 7:** Helical fraction determined based on far-UV CD spectra.

Peptide	Water	PBS	POPG		POPC	
			H_f_	Ө_222_/Ө_208_	H_f_	Ө_222_/Ө_208_
KR12-NH_2_	0.06	0.04	0.21	1.39	0.08	-
II	0.07	0.08	0.25	1.22	0.56	0.85
III	0.06	0.06	0.22	1.72	0.20	0.84
IV	0.06	0.07	0.23	1.27	0.15	0.64
V	0.08	0.07	0.17	1.94	0.15	0.61
VIII	0.06	0.07	0.46	1.07	0.27	0.79
XIII	0.08	0.11	0.49	1.11	0.54	0.84
XIV	0.06	0.06	0.35	1.14	0.27	0.82
XV	0.08	0.06	0.34	1.70	0.16	0.80
XVI	0.06	0.07	0.35	1.38	0.18	0.72
XVII	0.05	0.06	0.20	1.35	0.17	0.80
XVIII	0.08	0.07	0.49	1.05	0.44	0.82
XX	0.09	0.17	0.66	1.71	0.41	0.92
XXI	0.07	0.23	0.26	3.23	0.40	1.11
XXII	0.07	0.37	0.29	1.14	0.50	0.87

**Table 8:** Hydrodynamic diameter and zeta potential of POPG vesicles.

Sample	Peptide Concentration (mg/mL)	* D_H_ (nm)	Zeta Potential (mV)
POPG	-	113.8 ± 24.6	−36.7 ± 1.7
POPG/II	0.15	134.0 ± 28.9	−32.5 ± 1.6
	0.30	207.6 ± 72.9	−39.0 ± 1.9
POPG/IV	0.15	158.1 ± 48.7 549.4 ± 170.1	−31.3 ± 1.2
	0.30	163.7 ± 54.0 1078.6 ± 353.5	−28.2 ± 1.2
POPG/XIII	0.15	128.8 ± 32.2	−34.1 ± 1.5
	0.30	159.7 ± 57.7	−38.2 ± 1.5
POPG/XVIII	0.15	150.2 ± 47.5	−42.7 ± 1.9
	0.30	189.2 ± 63.25	−37.1 ± 1.8
POPG/XXI	0.15	174.6 ± 46.7	−37.9 ± 1.3
	0.30	239.8 ± 107.0	−31.3 ± 0.9

* The hydrodynamic diameters with standard deviation were obtained from the peaks with the highest scattered light intensity in light-scattering intensity distribution.

## Data Availability

Data are available upon reasonable request from the corresponding author.

## Supplementary Materials

The following supporting information can be downloaded at: https://www.mdpi.com/article/10.3390/ijms24065505/s1.

## Abbreviations

Boc, *tert*-butyloxycarbonyl; CD, circular dichroism; DCM, dichloromethane; DIC, *N*,*N*′-diisopropylcarbodiimide; DMF, *N*,*N*-dimethylformamide; DLS, dynamic light scattering; EDTA, ethylenediaminetetraacetic acid; ESI-MS, electrospray mass spectrometry; Fmoc, 9-fluorenylmethoxycarbonyl; LUVs, large unilamellar vesicles; Pbf, 2,2,4,6,7-pentamethyldihydrobenzofuran-5-sulfonyl residue; MIC, minimum inhibitory concentration; PBS, phosphate-buffered saline; POPC, 1-palmitoyl-2-oleoyl-glycero-3-phosphocholine; POPG, palmitoyl-2-oleoyl-sn-glycero-3-phosphoglycerol; RBCs, red blood cells; RP-HPLC, reversed-phase high-performance liquid chromatography; SPE, solid-phase extraction; TFA, trifluoroacetic acid; TIS, triisopropylsilane; Trt, trityl.
