# Supplementary material for: Influence of Lipidation Pattern of the KR12 Fragment of Peptide LL-37 on Its Antibacterial and Hemolytic Activities
Source: Int J Mol Sci. 2023 Mar 13;24(6):5505. doi: 10.3390/ijms24065505 (PMC10053710; doi:10.3390/ijms24065505)
Supplement: Supplementary file 1 [file ijms-24-05505-s001.zip › ijms-2262401-supplementary.pdf]

Supplementary Materials

Table S1. Results of ESI MS analyses of peptides I–XXII.

No.	Peptide	MS Analysis			No.	Peptide	MS Analysis		
		z	***m/z*** **calc.**	***m/z*** **found**			*z*	***m/z*** **calc.**	***m/z*** **found**
**I**	KR12-NH_2 (KRIVQRIKDFLR-NH_2)	1	1571.0	-	**XII**	C_8^{α}-retro-KR12-NH_2	1	1697.1	-
		2	786.0	786.6			2	849.1	849.5
		3	524.3	824.8			3	566.4	566.7
		4	393.5	-			4	425.0	425.6
							5	340.2	-
**II**	C_8^{α}-KR12-NH_2	1	1697.1	-	**XIII**	2-buthyloctanoic acid-KR12-NH_2	1	1753.2	-
		2	849.1	849.4			2	877.1	877.6
		3	566.4	566.8			3	585.1	585.5
		4	425.0	-			4	439.0	439.5
**III**	C_8^{ε}-KR12-NH_2	1	1697.1	-	**XIV**	2-ethylhexanoic acid-KR12-NH_2	1	1697.1	-
		2	849.1	849.6			2	849.1	849.6
		3	566.4	566.8			3	566.4	566.9
		4	425.0	-			4	425.0	425.4
**IV**	C_8^{α}-Lys-KR12-NH_2	1	1825.2	-	**XV**	Benzoic acid-KR12-NH_2	1	1675.0	-
		2	913.1	913.5			2	838.0	838.5
		3	609.1	609.5			3	559.0	559.5
		4	457.1	457.5			4	419.5	420.3
**V**	C_8^{ε}-Lys-KR12-NH_2	1	1825.2	-	**XVI**	Phenylacetic acid-KR12-NH_2	1	1689.0	-
		2	913.1	913.6			2	845.0	845.3
		3	609.1	609.7			3	563.7	564.2
		4	457.1	457.6			4	423.0	-
**VI**	KR12- $Lys^{\varepsilon}(C_8)$-NH_2	1	1825.2	-	**XVII**	3-Phenylpropionic acid- KR12-NH_2	1	1703.1	-
		2	913.1	913.6			2	852.0	852.5
		3	609.1	609.6			3	568.4	569.1
		4	457.1	457.7			4	426.5	427.1
**VII**	$[Lys^{\varepsilon}(C_8)]^{12}$KR12-$NH_2$	1	1669.1	-	**XVIII**	4-Phenylbutanoic acid-KR12-NH_2	1	1717.1	-
		2	835.0	835.5			2	859.0	859.5
		3	557.0	557.5			3	573.0	573.5
		4	418.0	-			4	430.0	430.6
**VIII**	$[Lys^{\varepsilon}(C_8)]^{8}$KR12-$NH_2$	1	1697.1	-	**XIX**	*trans*-cinnamic acid-KR12-NH_2	1	1701.0	-
		2	849.1	849.6			2	851.0	851.5
		3	566.4	566.8			3	567.7	568.1
		4	425.0	-			4	426.0	426.4
**IX**	C_8^{α},C_8^{ε}-KR12-NH_2	1	1823.2	-	**XX**	Phenylpropiolic acid-KR12-NH_2	1	1699.0	-
		2	912.1	912.7			2	850.0	850.4
		3	608.4	608.9			3	567.0	567.6
		4	456.6	-			4	425.5	-
**X**	C_4^{α}, C_4^{ε}-KR12-NH_2	1	1711.1	-	**XXI**	4-phenylbenzoic acid-KR12-NH_2	1	1751.1	-
		2	856.0	856.4			2	876.0	876.50
		3	571.0	571.6			3	584.4	584.8
		4	428.5	429.4			4	438.5	439.1
**XI**	retro-KR12-C_8^{ε}-NH_2	1	1697.1	-	**XXII**	Deoxycholic acid-KR12-NH_2	1	1945.3	-
		2	849.1	849.5			2	973.1	973.7
		3	566.4	566.9			3	649.1	649.6
		4	425.0	-			4	487.1	487.5

Table S2. Calculations of differences between hydrophobicity coefficients of terminal amino acid residues of KR12-NH_2 and its *retro*-analog.

Peptide	Sequence	Hydrophobicity Coefficients of Terminal Amino Acid Residues [1] (min)	Differences in Coefficients Between *Retro*-Analog and Parent Molecule	Final Effect (min) Δ = ΔLys + ΔArg
KR12-NH_2<br>*retro*-KR12-NH_2	KRIVQRIKDFLR-NH_2<br>RLFDKIRQVIRK-NH_2	*N*-terminal Lys: 1.3<br>*C*-terminal Lys: 0.0<br>*N*-terminal Arg: 3.0<br>*C*-terminal Arg: 3.7	ΔLys: −1.3<br>ΔArg: −0.7	−2.0
Ac-KR12-NH_2<br>Ac-*retro*-KR12-NH_2	Ac-KRIVQRIKDFLR-NH_2<br>Ac-RLFDKIRQVIRK-NH_2	*N*-terminal Lys: -0.3<br>*C*-terminal Lys: 0.0<br>*N*-terminal Arg: 2.4<br>*C*-terminal Arg: 3.7	ΔLys: 0.3<br>ΔArg: −1.3	−1.0

Hydrophobicity coefficients are from article of Tripet B *et al.* (2006) [1]. Previous studies on *retro*-analogs indicate that terminal amino acid residues are critical for change of retention behaviour and thus hydrophobicity [2]. Calculated differences in hydrophobicity coefficients of lysine and arginine between *retro*-analogs and parent molecules (KR12-NH_2, Ac-KR12-NH_2) indicate that both analogs will be more hydrophilic (negative numbers – "final effect").

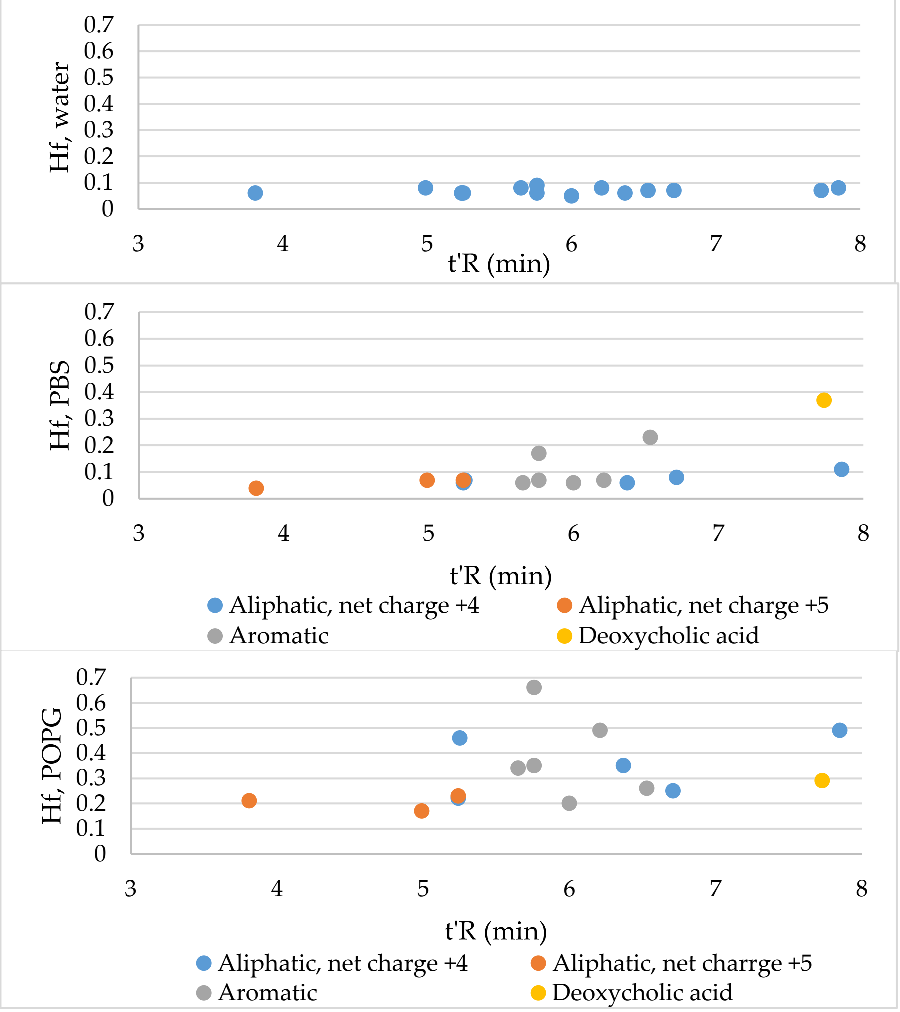

Figure S1. Helical fraction (Hf) *vs.* adjusted retention time.

References

1. Tripet, B.; Cepeniene, Dz.; Kovacs, J.M.; Mant, C.T. Oleg V. Krokhin b, Robert S. Hodges Requirements for prediction of peptide retention time in reversed-phase high-performance liquid chromatography: Hydrophilicity/hydrophobicity of side-chains at the N- and C-termini of peptides are dramatically affected by the end-groups and location. *J. Chromatogr. A* **2007**, *1141*, 212–225.

2. Neubauer, D.; Jaśkiewicz, M.; Migoń, D.; Bauer, M.; Sikora, K.; Sikorska, E.; Kamysz, E.; Kamysz, W. Retro analog concept: Comparative study on physico-chemical and biological properties of selected antimicrobial peptides. *Amino Acids* **2017**, *49*, 1755–1771.
